# Differences in Long-COVID Symptoms between Vaccinated and Non-Vaccinated (BNT162b2 Vaccine) Hospitalized COVID-19 Survivors Infected with the Delta Variant

**DOI:** 10.3390/vaccines10091481

**Published:** 2022-09-06

**Authors:** César Fernández-de-las-Peñas, Ricardo Ortega-Santiago, Stella Fuensalida-Novo, José D. Martín-Guerrero, Oscar J. Pellicer-Valero, Juan Torres-Macho

**Affiliations:** 1 Department of Physical Therapy, Occupational Therapy, Rehabilitation and Physical Medicine, Universidad Rey Juan Carlos, 28922 Alcorcón, Spain; 2 Intelligent Data Analysis Laboratory, Department of Electronic Engineering, ETSE (Engineering School), Universitat de València (UV), 46100 Valencia, Spain; 3 Department of Internal Medicine, Hospital Universitario Infanta Leonor-Virgen de la Torre, 28031 Madrid, Spain; 4 Department of Medicine, School of Medicine, Universidad Complutense de Madrid, 28040 Madrid, Spain

**Keywords:** COVID-19, vaccine, post-COVID, Delta, hospitalization

## Abstract

This study compared differences in the presence of post-COVID symptoms among vaccinated and non-vaccinated COVID-19 survivors requiring hospitalization due to the Delta (B.1.617.2) variant. This cohort study included hospitalized subjects who had survived SARS-CoV-2 infection (Delta variant) from July to August 2021 in an urban hospital in Madrid, Spain. Individuals were classified as vaccinated if they received full administration (i.e., two doses) of BNT162b2 (“Pfizer-BioNTech”) vaccines. Other vaccines were excluded. Those with just one dose of the BNT162b2 vaccine were considered as non-vaccinated. Patients were scheduled for a telephone interview at a follow-up around six months after infection for assessing the presence of post-COVID symptoms with particular attention to those symptoms starting after acute infection and hospitalization. Anxiety/depressive levels and sleep quality were likely assessed. Hospitalization and clinical data were collected from medical records. A total comprising 109 vaccinated and 92 non-vaccinated COVID-19 survivors was included. Vaccinated patients were older and presented a higher number of medical comorbidities, particular cardiorespiratory conditions, than non-vaccinated patients. No differences in COVID-19 onset symptoms at hospitalization and post-COVID symptoms six months after hospital discharge were found between vaccinated and non-vaccinated groups. No specific risk factor for any post-COVID symptom was identified in either group. This study observed that COVID-19 onset-associated symptoms and post-COVID symptoms six-months after hospitalization were similar between previously hospitalized COVID-19 survivors vaccinated and those non-vaccinated. Current data can be applied to the Delta variant and those vaccinated with BNT162b2 (Pfizer-BioNTech) vaccine.

## 1. Introduction

The quick spread of the severe acute respiratory syndrome coronavirus 2 (SARS-CoV-2), the agent provoking the coronavirus disease, 2019 (COVID-19), has changed the recent world. After the main worldwide outbreak leading to hundreds of millions of acute infections and almost seven million deaths, healthcare professionals are at the forefront of the post-COVID outbreak, which is the development or persistence of symptoms after the acute phase, a condition called long-COVID [1] or post-COVID-19 [2]. More than 100 symptoms affecting multiple systems, e.g., cardiovascular, neurological, musculoskeletal, have been described [3]. Several meta-analyses have reported that around 50% of COVID-19 survivors exhibit different post-COVID symptoms during the first six months after the infection [4,5] and one year after [6,7]. The presence of post-COVID symptomatology is associated with worse health-related quality of life [8].

An important step in the fight against the COVID-19 has been the development of vaccines. COVID-19 vaccines have significantly reduced the risk of developing the severe form of the disease and the mortality rate death caused by COVID-19 [9], however, they are not completely effective for preventing contagion and SARS-CoV-2 virus continues its spreading [10]. In fact, vaccinated people can be infected and suffer from asymptomatic, mild, or moderate COVID-19. Since long-COVID can arise even after a mild or asymptomatic SARS-CoV-2 infection [11], it has been questioned what impact the COVID-19 vaccines have on long-COVID [12,13,14,15].

Emerging evidence on the impact of vaccines on long-COVID is heterogeneous, but it seems that vaccines might only reduce minimally the risk of long-COVID or have no effect on it at all [16,17,18]. Two government summary reports summarized available evidence about the impact of vaccines on the risk of development long-COVID. However, analysis of the methodological quality of the literature was poor [19,20]. A systematic review has recently found a low level of evidence suggesting that vaccination before the infection could reduce the risk of the posterior development of long-COVID, but the impact of vaccines in people with existing long-COVID is still controversial [21]. This review has identified some pitfalls in the current literature: first, available data pooled data from hospitalized and non-hospitalized data together; second, no data about any specific SARS-CoV2 variant of concerns are present [21]. No study to date has specifically investigated the impact of vaccines in hospitalized COVID-19 survivors and controlling the SARS-CoV-2 variant of concern. Therefore, our main objective was to compare differences in the presence of post-COVID symptoms among vaccinated and non-vaccinated COVID-19 survivors requiring hospitalization due to the Delta (B.1.617.2) SARS-CoV-2 variant. A secondary aim was to identify potential risk factors associated with the development of post-COVID symptoms in vaccinated and non-vaccinated hospitalized survivors.

## 2. Methods

### 2.1. Participants 

This cohort study included subjects who were hospitalized due to their first acute SARS-CoV-2 infection from July to August 2021 in one urban hospital in Madrid, Spain. The diagnosis of SARS-CoV-2 infection was confirmed at hospital admission with real-time reverse transcription-polymerase chain reaction (RT-PCR) assay of nasopharyngeal and oral swab samples as well as presence of clinical and radiological findings. Sanger sequencing of the receptor binding domain (RBD) was used to determine the SARS-CoV-2 variant type. Only patients infected with the Delta (B.1.617.2) variant were included. The study was approved by the Local Ethic Committees of the Hospital (URJC0907202015920, HUIL/092-20,). All participants provided informed consent before collecting any data.

### 2.2. Procedure

Demographic (age, gender, height, weight), clinical (COVID-19 associated onset symptoms at hospital admission, pre-existing medical comorbidities), and hospitalization (intensive care unit -ICU- admission, days at hospital) data were collected from medical records. In addition, we also collected if the patient had received full dose of vaccination before the infection and hospitalization. Due to the presence of different vaccines, we just considered as vaccinated those participants with the full administration (i.e., two doses) of BNT162b2 (“Pfizer-BioNTech”) vaccine. Subjects vaccinated with other vaccines, e.g., AZD1222 (“Oxford-AstraZeneca”), mRNA-1273 (“Moderna”), and Ad26.COV2.S (“Janssen”) were excluded.

Participants who agreed to participate were scheduled for a telephone interview by trained researchers at a follow-up longer than six months after infection. Participants were asked to report the presence of symptoms appearing after hospital discharge and whether the symptoms persisted at the time of the study. Carefully attention was paid to the fact that symptoms should be attributable to COVID-19 and should have started no later than one month after acute infection and hospitalization. The following predefined list of post-COVID symptoms was systematically assessed: dyspnea, fatigue, anosmia, ageusia, hair loss, chest pain, palpitations, diarrhea, skin rashes, brain fog, ocular/visual disorders, cough, and loss of concentration. However, participants were free to report any symptom that they suffered from and considered relevant. 

The Hospital Anxiety and Depression Scale (HADS) and the Pittsburgh Sleep Quality Index (PSQI) were used for evaluating anxiety/depressive symptoms and sleep quality, respectively, since both can be properly assessed by telephone [22]. Both the anxiety (HADS-A, 7-items, 0–21 points) and depressive (HADS-D, 7-items, 0–21 points) scales were used [23]. A cut-off score of ≥12 points for the HADS-A ≥10 points for HADS-D were indicative of anxiety or depressive symptoms, respectively [24]. It has been recently found that the HADS exhibits good psychometric properties in individuals with long-COVID [25]. The PSQI (0–21 points) was used to assess sleep quality during the previous month, where a cut off of ≥8.0 points was considered indicative of poor sleeper [26]. 

### 2.3. Statistical Analysis

Data are presented as mean (standard deviation, SD) or number of cases (percentage) as appropriate. For the main aim, we compared the differences in post-COVID symptoms among vaccinated and non-vaccinated patients with Chi-squared for categorical variables or one-way-ANOVA tests for continuous variables. The level of significance was set at a priori 0.05 with *p*-values from all tests being corrected (Holm–Bonferroni correction). For the second aim, multivariate logistic regressions were conducted to identify potential associations of post-COVID symptoms with variables collected at the acute phase of the infection in either vaccinated or non-vaccinated patients, separately. Adjusted odds ratio (OR) and confidence intervals (95%CI) were calculated. Data were collected with STATA 16.1 and processed using Python’s library pandas 0.25.3. Scipy 1.5.2 was employed for conducting the statistical tests and statsmodels 0.11.0 for performing *p*-value correction. 

## 3. Results

From 300 hospitalized patients during the recruitment period, a total of 201 (mean age: 56.5, SD: 21 years, 54.4% women) patients fulfilled all criteria, agreed to participate and provided informed consent. The reasons for exclusion were: 1, infected with different SARS-CoV-2 variant (*n* = 45); 2, vaccinated with AZD1222 vaccine (*n* = 20); 3, vaccinated with Ad26.COV2.S (*n* = 12) vaccine; 4, refuse to participate (*n* = 12); or 5, death after hospital discharge (*n* = 10). From those participants included (*n* = 201), 54% (*n* = 109) formed the vaccinated group, whereas the remaining 46% (*n* = 92) formed the non-vaccinated group.

Table 1 summarizes clinical and hospitalization data in both vaccinated and non-vaccinated groups. Vaccinated patients were older and exhibited a higher prevalence of medical comorbidities (both, *p* < 0.001), particular cardiorespiratory conditions than non-vaccinated patients (Table 1). The most common symptoms associated with SARS-CoV-2 acute infection at hospital admission consisted of fever, dyspnea, myalgia, cough, and headache. No significant differences in COVID-19 onset-associated symptoms were found between vaccinated and non-vaccinated groups, except for a higher prevalence of anosmia within the non-vaccinated group (*p* = 0.02, Table 1). Finally, vaccinated patients needed longer hospital stay than non-vaccinated patients (*p* = 0.03, Table 1).

Participants were assessed a mean of 6.3 (SD 1.0) months after hospital discharge. Table 2 details post-COVID symptoms in both vaccinated and non-vaccinated groups. The most prevalent post-COVID symptoms 6 months after hospitalization were fatigue, dyspnea at exertion, pain symptoms, and memory loss. No significant differences in post-COVID symptoms between vaccinated and non-vaccinated groups were seen, except that a greater proportion of vaccinated patients reported dyspnea with exertion (*p* < 0.001) than non-vaccinated patients (Table 2). Additionally, vaccinated patients also exhibited worse sleep quality (*p* = 0.03) than non-vaccinated patients (Table 2).

The multivariate analysis did not reveal any significant risk factor associated with the development of post-COVID symptoms in neither vaccinated nor non-vaccinated group.

## 4. Discussion

This is the first study to date systematically investigating the development of post-COVID in vaccinated COVID-19 survivors who had need hospitalization and controlling the SARS-CoV-2 variant. We observed that COVID-19 onset-associated symptoms and post-COVID symptoms six-months after hospitalization were similar between previously hospitalized COVID-19 survivors fully vaccinated with BNT162b2 (“Pfizer-BioNTech”) vaccine and those non-vaccinated. 

We found that vaccinated patients were older and exhibited more previous medical comorbidities than non-vaccinated individuals. No differences in COVID-19 associated-onset symptoms at hospital admission (except for anosmia) were seen between vaccinated and non-vaccinated patients. The fact that vaccinated patients were older than those non-vaccinated would be an expected finding since vaccination strategies firstly focused on vulnerable, e.g., older, people. This strategy is based on current data suggesting that older individuals (>60 years) are at a higher risk of SARS-CoV-2 infection, but available data is not conclusive [27]. Similarly, older individuals are also at higher risk of severe COVID-19 complications even at a post vaccination status than younger individuals [28]. This age difference would explain higher number of previous medical comorbidities, particularly those related to the cardiorespiratory system, found in the vaccinated group. 

Importantly, albeit these differences in age and previous comorbidities between vaccinated and non-vaccinated groups, no overall differences in post-COVID symptoms at six months after hospitalization were observed. We just found that a higher number of vaccinated (older) patients reported dyspnea on exertion and worse sleep quality than non-vaccinated (younger) patients. Age differences could also explain these differences since older adults do not sleep as well as younger adults [29]. Further, a higher presence of cardiorespiratory medical comorbidities would also explain dyspnea on exertion, but not at rest within the vaccinated group. Interestingly, no significant sex differences were found between vaccinated and non-vaccinated groups, although a greater proportion of female were vaccinated. Female sex has been identified as another risk factor for post-COVID symptoms [27]. In our study, sex differences, such as older age, were not determinant for our results. 

Although some studies have proposed that older age and previous comorbidities are risk factors for developing post-COVID symptoms [30,31,32], data from meta-analyses are not conclusive in this assumption [27,33]. The results of our study would agree with the results from these meta-analyses [27,33] since no differences in post-COVID symptoms were seen between groups albeit age and comorbidities differences. The most important finding was the lack of differences between groups considering that one group was fully vaccinated with BNT162b2 (“Pfizer-BioNTech”) vaccine and the other did not. Current results disagree with those recently found by the review by Notarte et al. [21] where studies evaluating the impact of vaccination before the acute infection showed a trend towards a decreased risk of developing long-COVID symptomatology. Discrepancies could be related to the fact that we only included hospitalized patients (previous studies pooled hospitalized and non-hospitalized individuals together) and that we just included subjects infected with the Delta variant (previous studies did not specify the variant). Another potential explanation for discrepancies is that the impact of vaccination prior to SARS-CoV-2 acute infection on long-COVID symptoms is marked in COVID-19 survivors younger than 60 years-old, whereas no clear impact has been observed in those older than 60 years-old [34]. Finally, we just considered the impact of the BNT162b2 (“Pfizer-BioNTech”) vaccine, whereas previous studies have mixed results from different types of vaccines [21]. 

Two hypotheses are proposed for potential underlying mechanisms explaining the effects of vaccination on the risk of developing future post-COVID. First, since vaccines reduce the severity of COVID-19 disease, this lower severity of the condition may then translate into a lower risk of developing long-COVID symptoms. The meta-analysis by Maglietta et al. [27] reported that acute disease severity was a risk factor for developing long-COVID symptomatology. A second potential hypothesis would be that the vaccine may accelerate clearance of remnant SARS-CoV-2 virus in the human body (viral persistence hypothesis) [35] and, hence, also reduce the exaggerated inflammatory-immune response associated with this virus persistence (immune/inflammatory hypothesis). Future studies investigating these hypotheses would be needed.

Although this is the first study investigating the impact of COVID-19 vaccine in a controlled sample of hospitalized patients, current data should be considered according to their limitations. First, these results should be only applicable to previously hospitalized COVID-19 survivors infected with the Delta variant and vaccinated with the BNT162b2 (“Pfizer-BioNTech”) vaccine. In addition, our sample size is limited comparing with other studies on the same topic (21). Accordingly, extrapolation of current results to the general COVID-19 population is limited. Future studies including larger populations recruited from different hospitals will support the generalization of current results. Second, we did not collect COVID-19 severity or serological biomarkers of inflammation at hospital admission or follow-up, which could help to elucidate differences in these variables between vaccinated and non-vaccinated individuals. Third, the cross-sectional design of the study does not allow to determine the evolution of post-COVID symptoms, making it difficult to exclusively attribute to SARS-CoV-2 the presence of symptoms at six months after hospitalization. Fourth, we assigned individuals to the vaccinated group if they had received the full (two) dose of the vaccine. In fact, individuals who had received just one dose were considered as non-vaccinated. Finally, we evaluated the impact of COVID-19 vaccine when administered before the infection. Other studies have evaluated the impact of COVID-19 vaccines in people with ongoing current long-COVID symptoms [36].

## 5. Conclusions

The current study observed no overall differences in COVID-19 onset-associated symptoms and post-COVID symptomatology six months after hospitalization between vaccinated and non-vaccinated (BNT162b2 “Pfizer-BioNTech”) previously hospitalized COVID-19 survivors infected with the Delta variant. 

## Figures and Tables

**Table 1:** Clinical and Hospitalization Data according to Vaccine Status.

	Vaccinated (*n* = 109)	Non-Vaccinated (*n* = 92)	*p*-Value
Female (n, %)	67 (61.5%)	43 (46.75%)	0.159
Age (years) *	65.0 ± 21.0	46.5 ± 16.0	<0.001
Weight (kg)	77.7 ± 13.5	76.6 ± 13.9	0.557
Height (cm)	164.8 ± 8.6	167.5 ± 9.2	0.304
Previous Medical Co-morbidities			
Number of pre-existing co-morbidities *	1.7 ± 1.0	0.85 ± 0.95	<0.001
Obesity (pre-existing)	33 (30.3%)	20 (21.7%)	0.240
Hypertension (pre-existing)	51 (46.8%)	21 (22.8%)	0.318
Diabetes (pre-existing)	23 (21.1%)	5 (5.4%)	0.212
Asthma (pre-existing)	12 (11.0%)	7 (7.6%)	0.434
COPD (pre-existing) *	10 (9.2%)	2 (2.2%)	0.04
Pain (pre-existing) *	51 (46.8%)	26 (28.25%)	0.03
Cardiac diseases (pre-existing) *	21 (19.3%)	6 (6.5%)	0.01
Rheumatological diseases (pre-existing) *	29 (26.6%)	6 (6.5%)	<0.001
Other diseases (pre-existing)	22 (20.2%)	13 (14.1%)	0.305
COVID-19 associated-onset symptoms at hospital admission			
Number of COVID-19 symptoms	2.4 ± 0.9	2.65 ± 0.65	0.677
Fever (COVID-19 onset)	70 (64.2%)	60 (65.2%)	0.930
Dyspnea (COVID-19 onset)	22 (20.2%)	27 (29.35%)	0.189
Myalgias (COVID-19 onset)	39 (35.8%)	29 (31.5%)	0.605
Cough (COVID-19 onset)	37 (33.9%)	30 (32.6%)	0.870
Headache (COVID-19 onset)	40 (36.7%)	26 (28.25%)	0.298
Diarrhea (COVID-19 onset)	4 (3.7%)	13 (14.1%)	0.708
Anosmia (COVID-19 onset)	15 (13.8%)	14 (15.2%)	0.786
Ageusia (COVID-19 onset) *	11 (10.1%)	21 (22.8%)	0.02
Throat pain (COVID-19 onset)	15 (13.8%)	17 (18.5%)	0.404
Vomiting (COVID-19 onset)	1 (0.9%)	2 (2.2%)	0.467
Dizziness (COVID-19 onset)	5 (4.6%)	6 (6.5%)	0.559
Days at hospital *	12.9 ± 12.45	9.7 ± 7.8	0.03
ICU admission	11 (10.1%)	8 (8.7%)	0.748

* Significant differences between vaccinated and non-vaccinated groups (*p* < 0.05).

**Table 2:** Post-COVID Symptoms and Psychological Symptoms according to Vaccine Status.

	Vaccinated (*n* = 109)	Non-Vaccinated (*n* = 92)	*p*-Value
Number of post-COVID symptoms	2.5 ± 1.1	2.5 ± 1.3	0.757
Post-COVID Symptomatology			
Fatigue	86 (78.9%)	68 (73.9%)	0.687
Dyspnea at exertion *	93 (85.3%)	16 (17.4%)	<0.001
Pain Symptoms (excluding headache)	44 (40.4%)	39 (42.4%)	0.823
Memory Loss	22 (20.2%)	14 (15.2%)	0.407
Hair Loss	33 (30.3%)	40 (43.5%)	0.122
Headache	14 (12.9%)	17 (18.5%)	0.310
Dyspnea at rest	18 (16.5%)	8 (8.9%)	0.252
Gastrointestinal Problems	16 (14.7%)	12 (13.05%)	0.756
Cognitive Blurring-Frain Fog	12 (11.0%)	10 (10.9%)	0.976
Throat Pain	11 (10.1%)	7 (7.6%)	0.558
Skin Rashes	6 (5.5%)	4 (4.35%)	0.714
Ageusia	6 (5.5%)	4 (4.35%)	0.714
Anosmia	9 (8.25%)	3 (3.25%)	0.149
Ocular Problems	7 (6.4%)	2 (2.2%)	0.156
Palpitations-Tachycardia	2 (1.8%)	6 (6.5%)	0.101
Concentration Loss	2 (1.8%)	4 (4.35%)	0.304
Voice Problems	2 (1.8%)	2 (2.2%)	0.865
Psychological and Emotional Symptoms			
HADS-A (0–21)	3.5 ± 3.2	2.5 ± 3.0	0.245
Anxiety (HADS-A ≥12 points)	2 (1.8%)	1 (1.1%)	0.902
HADS-D (0–21)	3.8 ± 3.3	2.5 ± 3.3	0.499
Depression (HADS-D ≥10 points)	5 (4.6%)	5 (5.4%)	0.891
Sleep Quality (0–21) *	7.6 ± 3.45	6.5 ± 3.7	0.025
Poor Sleep Quality (PSQI ≥8 points) *	51 (46.8%)	27 (29.35%)	0.03

* Significant differences between vaccinated and non-vaccinated groups (*p* < 0.05).

## Data Availability

Not applicable.
